# A Randomized Controlled Trial of Paula Method Versus Gum Chewing for Gastrointestinal Reactivation After Cesarean Delivery

**DOI:** 10.3390/jcm15031205

**Published:** 2026-02-03

**Authors:** Nadezda Koryakina, Amy Solnica, Michal Liebergall Wischnitzer, Wiessam Abu Ahmad, Joshua Isaac Rosenbloom

**Affiliations:** 1 Henrietta Szold Hadassah University Medical Center School of Nursing, Faculty of Medicine, Hebrew University of Jerusalem Kalman Ya'Akov Man Street, Jerusalem 9112000, Israel; 2 Braun School of Public Health, Hebrew University-Hadassah, Kalman Ya'Akov Man Street, Jerusalem 9112000, Israel; 3 Branch of Planning and Strategy, Clalit Health Services, 101 Arlozorov St., Tel Aviv 6209804, Israel; 4 Department of Obstetrics and Gynecology, Hadassah University Medical Center, Faculty of Medicine, Hebrew University of Jerusalem, Kalman Ya'Akov Man Street, Jerusalem 9112000, Israel

**Keywords:** cesarean delivery, gastrointestinal recovery, Paula method exercises, nursing

## Abstract

**Background*/*Objective***:* Women after cesarean delivery (CD) may feel discomfort and pain until the gastrointestinal (GI) activation. Standard care approaches following an elective cesarean delivery often fail to address the needs of patients. Nurses care for women after CD, managing their symptoms and promoting GI activity to prevent ileus. Randomized controlled trials (RCTs) have shown that gum chewing is an effective method compared to standard care. Additionally, pilot RCTs have found Paula method exercises to be beneficial in comparison to standard care. This study aims to compare the time of first flatus and first defecation between the Paula method group and the gum-chewing group in women after an elective CD. **Methods:** A randomized controlled trial was conducted with 90 women; forty-four women were randomized to the Paula method exercises, and forty-six to gum chewing. Both groups received standard care. The primary outcomes were the time to the passage of the first flatus and the time to the first defecation from the delivery. **Results:** There was no significant difference between groups in time to flatus or time to defecation, yet there was a median 8.2 h shortening of time to flatus in the Paula group (19.7 h [IQR 15.7–28.3] in the Paula group versus 27.9 h [IQR 17.6–38.2] in the gum-chewing group). In an exploratory analysis of the first 16 h post-cesarean delivery, the gum-chewing group showed a shorter time to passage of the first flatus compared to the Paula group. **Conclusions:** Gum chewing is recommended as part of the current guidelines to enhance recovery after surgery, yet it is not suitable for all. While the Paula method appears safe and demonstrates promise, definitive conclusions require validation from larger, adequately powered trials.

## 1. Introduction

Cesarean delivery (CD) prevalence has steadily increased over recent decades, with 21.1% of women delivering by CD worldwide [1]. Geographic rates vary significantly, from 5% in Sub-Saharan Africa to an average of 42.8% in Latin America and the Caribbean, with projections indicating a global CD rate of 28.5% by 2030 [1]. CD can result in various postoperative complications, such as pain, discomfort, and impaired peristalsis, potentially leading to paralytic ileus, a temporary cessation of bowel activity [2]. Liu and colleagues [3] found that the rates of abdominal distension and intestinal obstruction were 15.1% and 0.7%, respectively. Postoperative ileus is associated with symptoms such as discomfort, nausea, vomiting, abdominal distension, abdominal pain, and obstipation [4]. Potential risk factors for postoperative ileus include exposure to general anesthesia, opioid analgesia, a diagnosis of preeclampsia, significant blood loss, and the transfusion of blood products [2,5].

Gastrointestinal (GI) function recovery after CD remains a health issue [6], as 75% of women post-CD describe several symptoms, including lower back pain, headaches, and pain localized to the CD site [7]. Furthermore, CD is linked to moderate to severe postpartum pain [8]. Nearly all experience abdominal pain and discomfort following a CD, which can be alleviated through various interventions [9]. Nearly 42% of patients experience mild ileus symptoms, such as nausea, vomiting, and bloating postpartum, regardless of when feeding begins after CD [10]. A considerable number of women also report abdominal distension, with some of them developing abdominal obstruction following the CD [3]. Postoperative impairment of GI function may influence the length of hospitalization [11].

Ileus prevention is part of the clinical guidelines after a CD and routine postpartum treatment [12]. To prevent the development of an ileus, the Enhanced Recovery After Surgery (ERAS) guidelines recommend early oral feeding, early ambulation, and removal of the urinary catheter. They also recommend reducing IV fluids and opioid use and implementing multimodal analgesia [13]. Gum chewing is included in the ERAS protocol following a CD [14,15]. The outcomes for ileus resolution are the presence of bowel sounds, passage of flatus, and defecation [16,17]. Vagal activity, which plays a crucial role in maintaining digestion, may be severely reduced by surgery [18]. Thus, vagus nerve stimulation was found to improve the postoperative restoration of gastrointestinal motility [19]. Standard postpartum care does not always align with international clinical guidelines [20]. Although international experts engaged in the Delphi process advise against using laxatives to prevent postoperative ileus, they continue to be utilized in post-cesarean care [21].

Gum chewing has been linked to a faster recovery of GI function following surgery [22] and may provide non-specific stimulation of the GI system [23]. Yet, its effectiveness has been doubted after the introduction of early feeding into post-surgery recovery protocols [11]. In a RCT, Hassan and colleagues [24] found that the gum-chewing group had a shorter time to first flatus passage compared to the early mobilization, early hydration, and standard groups after CD. According to a systematic review and meta-analysis, gum chewing was found to significantly reduce, among others, the time until the first flatus (MD = −6.49 h, [95% confidence interval (CI) −8.65 to −4.33], first bowel sounds, and lead to less incidence of ileus and fewer episodes of nausea or vomiting [25]. The time to the first flatus was significantly shortened in the xylitol gum-chewing group compared to the control group (17.35 [6.27] vs. 11.18 [5.39] h, respectively, *p* = 0.003) [26]. No significant difference was found in the time to the first flatus and first defecation post-CD among the gum-chewing, coffee-drinking, and control groups in a RCT [27]. Gum chewing did not decrease the incidence of GI symptoms such as nausea and vomiting after an elective CD [28].

The Paula method exercises are based on the principle that all circular muscles in the body are interconnected. As a result, contracting and releasing one circular muscle naturally activates other muscles, including those in the GI system [29]. A RCT showed that this method, compared to standard care, reduced the time to the first passage of flatus following an elective CD compared to the standard group (mean 24.07 [6.85] h vs. 39.07 [10.37] h, respectively, *p* ˂ 0.001) [30]. Building on this study and the observed postoperative GI reactivation associated with gum chewing, the investigators planned to compare the Paula method exercises to gum chewing for GI system reactivation post-CD. Since standard care may not necessarily be effective in preventing paralytic ileus and may not be suitable for all women after a CD, alternative approaches are needed to accelerate the recovery of bowel function. The aim of this study is to compare the time of first flatus and first defecation between the Paula method group and the gum-chewing group in women after an elective CD.

## 2. Materials and Methods

### 2.1. Study Design

This randomized, controlled, non-blinded trial compared the Paula method and exercise with gum chewing for GI reactivation in women post-elective CD. Recruitment occurred between 20 February and 22 May 2023. The study was registered at Clinicaltrials.gov (NCT05729984) and adhered to the EQUATOR guidelines and CONSORT guidelines [31].

### 2.2. Setting and Sampling

The study population consisted of a convenience sample of women undergoing elective CD, either primary or repeat, who were hospitalized at a single, tertiary medical center and were randomly assigned to either the Paula method exercises group or the gum-chewing group.

Based on prior data indicating that the mean time to first flatus following the Paula treatment is 24.1 h [30], we estimated the required sample size to detect a clinically meaningful difference between the gum-chewing group. We assumed a standard deviation of 10 h and that the Paula method would reduce the time to flatus compared to gum chewing by 6.23 h [25,32,33]. Assuming a two-sided alpha of 0.05, and 80% power, we estimated that we needed 41 patients in each group. To account for possible dropout, we increased the sample size by 10% to arrive at a final size of 45 women in each group. The study inclusion criteria were women over the age of 18 years after an elective CD who could read and write in Hebrew. Excluded were those with chronic diseases of the GI system, inability to chew gum, regular laxative use, anxiety, or a history of postpartum depression. However, women who required a laxative or glycerin suppository as a “one-off” event after the first flatus but before the first bowel movement were excluded from the time to first bowel movement analysis. Women were recruited during the preoperative visit, approximately one week before the elective CD. At this visit, they received an explanation of the study, including the two interventions, and signed the informed consent form.

### 2.3. Interventions

All participants provided written informed consent. Randomization into either the Paula method group or the gum-chewing group was performed using a table of random numbers with sequentially numbered, opaque, and sealed envelopes prepared in advance by the nurse researcher (N.K.). Participants received their group allocation upon admission to the postpartum department, approximately two hours after the CD. The nurse researcher provided the women assigned to the Paula method exercise group with detailed verbal and written explanations of the exercises, along with illustrations [30], and instructed them to begin the exercises and practice them six times daily, each session lasting five minutes.

Women assigned to the gum-chewing group were provided with a package of sugar-free gum and instructed to chew it three times a day for 30 min each time [33] for the duration of their hospitalization, beginning upon admission to the postpartum unit.

All participants received the standard nursing care provided after an elective CD. This care included encouraging early eating and drinking (around 2 h after the CD), getting out of bed 8 h after birth, and encouraging ambulation. This standard nursing treatment is intended to, among other things, prevent complications of CD, including an ileus. If after 48 h from the CD the parturient did not pass the first flatus or defecation, she was able to receive laxatives or glycerin suppositories. Notably, gum chewing was not included in the standard care provided to women after CD in the postpartum unit.

### 2.4. Study Instruments

The study utilized two instruments. A demographic and health information tool was developed for this study to document information obtained from the participants’ medical records. Data included the number of pregnancies, number of births, reason for the elective CD, body mass index (BMI), date and time of the delivery, and type of anesthesia. In addition, a self-report form was created for the participants to document the group allocation (Paula method or gum chewing), frequency of intervention, and date and time of the first passage of flatus and the first defecation. The tool also contained questions about the use of other aids to promote GI activation, such as glycerin suppositories, enemas, laxatives, etc.

### 2.5. Data Collection and Data Analysis

Both groups were instructed on completing the self-report form, documenting the Paula method exercises and the gum chewing respectively, as well as reporting the passage of the first flatus and defecation. The time from delivery to the first flatus and the first defecation were calculated.

The data were entered into an Excel spreadsheet and then uploaded to R Studio for statistical analysis (version 2024.12.1). Pearson’s chi-squared test, Fisher’s exact test, and Welch’s two-sample *t*-test were used, as appropriate, to compare demographic and health variables between the groups. Time to the first passage of flatus and the first defecation were compared between groups using median and interquartile range (IQR) due to the non-normality of the data. We also performed a secondary time to event analysis. Kaplan–Meier survival curves were generated to examine the relationship between the intervention groups and the time to the first passage of flatus and first defecation. After noting that there was a change in the survival curves at 16 h, we fitted two separate Cox proportional hazards models for passage of first flatus, one for the early follow-up period (≤16 h) and another for the late follow-up period (>16 h). This approach allowed estimation of group effects in a time-specific manner, without assuming a constant hazard ratio across the entire follow-up period and without causing violation of the proportional hazards assumption. The decision to stratify the analysis at 16 h was informed by visual inspection of the Kaplan–Meier survival curve, which suggested a change in hazard patterns around that time point. Statistical significance was determined using two-tailed tests, with *p*-values < 0.05 considered significant.

### 2.6. Ethical Considerations

The study was approved by the medical center’s Helsinki Committee (Approval number 0491-22-HMO, 15 January 2023). All participants provided written consent at the preoperative visit.

## 3. Results

**Figure 1:**
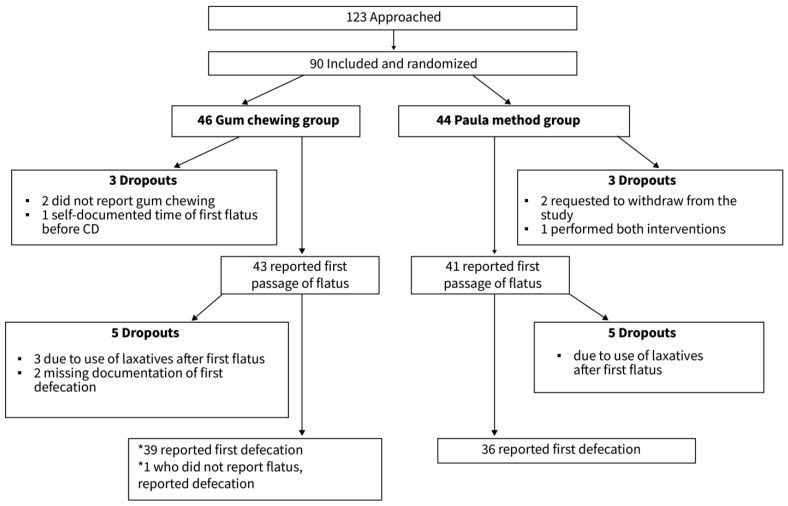
Study flowchart.

One hundred and twenty-three women were approached for participation. Thirty-three (27%) refused to participate, and 90 (73%) were included. The participants were divided into two groups: 44 (48.9%) in the Paula method exercises group and 46 (51.1%) in the gum-chewing group. In both groups, some women withdrew from the study at varying follow-up times (Figure 1).

The demographic and health variables of the randomized women did not show significant differences between groups, except for the indication for CD, *p* = 0.013. (Table 1). None of the patients had a blood transfusion or a diagnosis of preeclampsia, or any other intra- and postpartum complication. Five women in the Paula group and nine in the gum-chewing group received opioids, with no significant difference between the groups. All women received spinal anesthesia except one woman in each group, who received a combination of spinal and epidural anesthesia, and all were planned, scheduled CDs.

**Figure 2:**
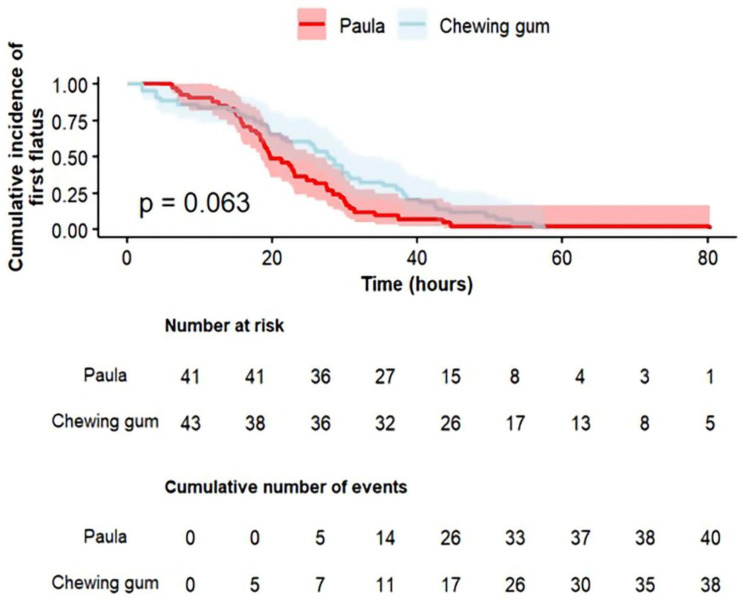
Kaplan–Meier survival curve of the association between the intervention group and the time (in hours) from the CD until the first flatus passage.

**Figure 3:**
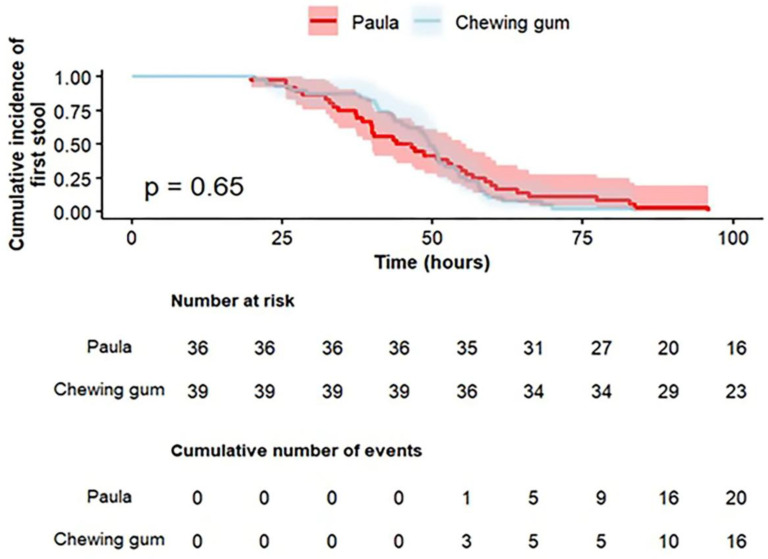
Kaplan–Meier survival curve of the association between the intervention group and the time (in hours) from the CD until the first defecation.

The median and the IQR of the first passage of flatus post-CD were shorter by 8.2 h in the Paula method exercises group, and median 19.7 h (IQR 15.7–28.3) versus median 27.9 h (IQR 17.6–38.2) in the gum-chewing group; but, this did not reach statistical significance (*p* = 0.079). The median and the IQR of the first defecation post-CD were similar between the groups, with a median of 45.3 h (IQR 35.8–57.8) in the Paula method group and a median of 49.6 h (IQR 41.1–55.6) in the gum-chewing group (*p* = 0.532). The findings are further illustrated in a secondary time-to-event analysis using Kaplan–Meier survival curves (Figure 2 and Figure 3). Visual inspection of the survival curve for time to the first flatus (Figure 2) revealed a clear crossing between the groups at 16 h post-CD (*p* = 0.063).

In the time-specific analysis, the log-rank test was significant during the first 16 h (*p* = 0.032), but not beyond 16 h (*p* = 0.078); however, this analysis was post hoc and therefore results should be interpreted with caution. At this early interval, the gum-chewing group demonstrated a significantly shorter time to passage of the first flatus compared to the Paula method group.

## 4. Discussion

This RCT aimed to compare the Paula method to gum chewing for GI reactivation post-elective CD; another three-armed RCT comparing the Paula method, gum chewing, and standard care. The authors reported that both the Paula method and gum chewing were significantly effective in GI reactivation in women post-CD [34].

The current study found a median difference of 8.2 h between the groups in time to first flatus. This clinically meaningful finding did not reach statistical significance. The groups had similar times to the first defecation post-CD. It is important to note that the study relied on patient self-report, as is customary in the evaluation of passage of first flatus and defecation post-surgery [25]. Self-report outcome measures may be subject to variation in reporting by the subjects. A previous RCT comparing the Paula method exercises to standard care in women following elective CD reported a significant advantage for the intervention group in the time to first flatus (24.1 [6.85] h vs. 39.1 [10.4] h, respectively, *p* < 0.001) [30]. In the present study, the Paula method exercises were compared to gum chewing, as this intervention has been incorporated into clinical guidelines for post-CD care [6]. The variation in results may be explained by the current study’s comparison of two interventions (Paula method exercises and gum chewing), while the previous study employed standard care for the control group. A recent RCT by Brundha et al. [35] reported a significant difference between a gum-chewing group and a control group following elective or emergency CD. The mean time to the first flatus after surgery in the gum-chewing group was 12.74 h (SD 4.841), and 20.51 h (SD 5.192) in the control group, *p* = 0.0001. The mean time to the first defecation was 41.59 h (SD 12.351) in the gum-chewing group and 64.03 h (SD 33.413) in the control group, *p* = 0.0001. The mean time until the first flatus was shorter in both groups compared to the current study. Similarly, the mean time until the first defecation was shorter in the intervention group and longer in the control group compared to the current study. This difference may be attributed to the younger age of participants in Brundha and colleagues’ RCT, with a mean age of 24.52 years (SD 3.93), compared to the older mean age of participants in the current study. The difference in time to first defecation between the control group in Brundha and colleagues’ [35] RCT and the current study may be explained by the fact that both groups in the current study received an additional intervention aimed at enhancing GI recovery. Another RCT involving 470 primipara women following elective CD evaluated the effects of acupressure, gum chewing, coffee consumption, and standard care on the time to first flatus and defecation. The acupressure group demonstrated a significantly shorter time to both first flatus and first defecation compared to all other groups (median 1447.5 min [540–2820, min–max], and 1485 min [1200–2025], respectively, *p* ˂ 0.001) The study found no significant differences between the gum-chewing, coffee-drinking, and control groups in terms of the time until the first flatus and the first stool [27]. Notably, in the RCT by Kanza Gül and Şolt Kırca [27], the gum-chewing group experienced a longer time to first flatus than observed in the current study, yet a shorter time to first defecation. It is important to note that the study populations differed between the two studies, particularly in parity and in the types of interventions provided.

Overall, there was no significant difference between the groups in the time to the first flatus. However, when exploring the first 16 h post-CD, gum chewing was found to significantly reduce the time to first flatus compared to the Paula method. It is important to note that this analysis was not planned *a priori*. This suggests that the effect of the interventions may not be constant over time and could differ between early and late postoperative periods. Furthermore, the Paula method is unfamiliar, and there is a “learning curve” to the performance of the exercises.

## 5. Strengths and Limitations

The current RCT study explored the use of the Paula method compared to gum chewing, included in the ERAS, for GI reactivation post-CD. Limitations can be related to recruitment from a single hospital, which can limit the generalizability of the findings to more diverse populations. Secondly, the study utilized a non-blinded design, due to the essence of the study interventions, which could have a potential impact on the self-reported outcome measures. Thirdly, adherence to the exercise and gum chewing was not fully monitored. Another limitation is that we may have been underpowered to detect small clinically meaningful differences. In addition, due to the lack of literature directly comparing these two interventions, the trial was powered to compare Paula vs. standard of care and gum vs. standard of care—not directly for Paula vs. gum chewing. A non-inferiority design may have been more suitable.

## 6. Conclusions

This study aimed to compare the Paula method to gum chewing for GI reactivation post-CD. Overall, no statistically significant differences were found between the groups. While the Paula method appears safe and demonstrates promise as a subject for future inquiry, definitive conclusions regarding its comparative effectiveness or clinical integration require validation from larger, adequately powered trials. Regarding time to first flatus, gum chewing was found to be more effective in the first 16 h in an exploratory analysis. Future studies should consider combining gum chewing and the Paula method, especially in the early postoperative period. Beyond women recovering from elective cesarean delivery, the Paula method should be explored in patients recovering from various surgeries, including emergency CD, and particularly among populations for whom gum chewing is unsuitable due to dental issues, cultural restrictions, or other factors.

## Figures and Tables

**Table 1:** Demographic and health data (N = 90).

Variable	Paula N = 46	Gum Chewing N = 44	*p* ^
Age, years Mean ± SD	32.82 ± 5.70	32.41 ± 5.67	0.736
BMI (kg/m^2^) Mean ± SD *	32.19 ± 5.42	31.0 ± 5.38	0.334
Number of Pregnancies Mean ± SD	4.45 ± 2.74	4.22 ± 2.53	0.671
Number of Deliveries Mean ± SD	2.86 ± 2.22	2.67 ± 2.24	0.687
Gestational week Mean ± SD	37.81 ± 1.29	37.78 ± 0.84	0.956
Number of past CD Mean ± SD	1.71 (1.82)	1.45 (1.41)	0.428
Delivery < 37 weeks n (%)	4 (9.1)	2 (4.3)	0.429
Indication for the CD *n* (%)			0.013
Past CD	23 (52)	33 (72)	
Past OASI’s	9 (20)	1 (2.2)	
Other	12 (27)	12 (26)	

* BMI available for 79 participants ^ Welch’s two-sample *t*-test; Fisher’s exact test; Pearson’s chi-squared test.

## Data Availability

Deidentified data will be available upon reasonable request.
